# Axillary sentinel lymph node biopsy after mastectomy: a case report

**DOI:** 10.1186/1477-7819-8-59

**Published:** 2010-07-09

**Authors:** Diego A Vicente, Leonard R Henry, George Hahm, Peter W Soballe, DeeDee Smart

**Affiliations:** 1 Department of Surgery, National Naval Medical Center, Bethesda, MD, USA; 2 Uniformed Services University of the Health Sciences, Bethesda, MD, USA; 3 Department of Surgery, Naval Medical Center San Diego, San Diego CA, USA; 4 Radiation Oncology Branch, National Cancer Institute, Bethesda, MD, USA

## Abstract

**Background:**

Sentinel lymph node biopsy has been established as the preferred method for staging early breast cancer. A prior history of mastectomy is felt to be a contraindication.

**Case presentation:**

A patient with recurrent breast cancer in her skin flap was discovered to have positive axillary sentinel nodes by sentinel lymph node biopsy five years after mastectomy for ductal carcinoma in situ.

**Conclusion:**

A prior history of mastectomy may not be an absolute contraindication to sentinel lymph node biopsy.

## Background

Sentinel lymph node (SLN) biopsy affords staging accuracy equal to that of complete axillary dissection [1] with reduced morbidity [2]. As such, it has become the preferred staging method for most patients with breast cancer [3]. A previous mastectomy has long been considered a prohibitive factor for reliable SLN biopsy in cases of recurrent cancer. We report the fifth patient, previously treated with mastectomy, found to have metastatic breast cancer in an axillary SLN biopsy performed at the time of cancer recurrence.

## Case presentation

A 41 year old female with DCIS of the left breast was treated at an outside hospital in 2002 with a total mastectomy. Tamoxifen was not administered. Implant based reconstruction was accomplished. In 2007 she was referred to our center with a 5 mm red nodule of the skin above her left breast incision scar with a palpable density beneath. There were no palpable axillary lymph nodes. A biopsy of the lesion demonstrated ER positive, HER2/neu negative invasive breast cancer.

**Figure 1:**
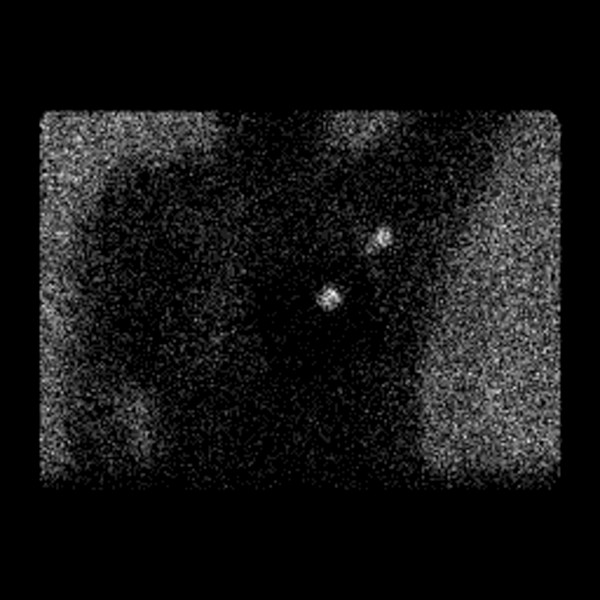
**Lymphoscintigraphy**. Increased uptake demonstrated at site of injection and at sentinel nodes.

On the morning of surgery, preoperative lymphoscintigraphy was performed with injection of 250 microcuries in the surrounding dermal component of the recurrent tumor. Immediate and fifteen minutes lymphoscintigraphy with lead shield at injection site mapped to the ipsilateral axillary nodal basin (Figure 1). Therefore, it was felt that an attempt at SLN biopsy would be reasonable. Intra-operatively, 1 mL of lymphazurin was also injected in the dermis in four quadrants surrounding the tumor. Three SLNs were identified, all of which were blue and had increased radioactivity.

The recurrent tumor was widely excised and the implant removed. Pathology revealed a 1.3 cm, ER positive invasive breast cancer with three of three SLNs involved with metastatic cancer. Subsequently, the patient received adjuvant chemotherapy with doxorubicin, cyclophosphamide, and docetaxel, after which a completion axillary lymph node dissection removed an additional 20 nodes, which were all negative for malignancy. A total of 50.4 Gy external beam radiotherapy was administered to the left chest wall, axilla, and supraclavicular areas. The patient has been receiving exemestane and has been disease free for 33 months.

## Discussion

SLN biopsy for breast cancer was rapidly assimilated into mainstream practice shortly after its introduction by *Giuliano et al. *[4]. Consensus statements, such as the Philadelphia conference [5], set relatively strict guidelines to help institute quality control and to ensure low false negative rates for this relatively new method of axillary staging. As experience and comfort with SLN biopsy has grown, studies have demonstrated feasibility and accuracy in staging the axilla in many clinical situations initially felt to represent contraindications [6-11].

The breast has an organized lymphatic pathway through which breast cancer typically follows lymph from its primary location in the lobule into subareolar plexus [12] and then to common lymphatic channels leading to the axillary nodes and less commonly to the internal mammary nodes or other surrounding sites [13,14]. A previous breast treatment with mass excision and axillary surgery was at first thought to significantly disrupt these lymphatic pathways. Several studies, however, have demonstrated that SLN biopsy remains feasible [15-19], but there is a decreased identification rate of SLN and an increased incidence of lymphatic drainage to SLN outside of the ipsilateral axilla in this setting.

Similarly, a previous mastectomy should profoundly interrupt the lymphatic pathway, and as such has been held to be one of the last contraindications to SLN biopsy in breast cancer. Our case adds to a growing literature base that suggests that even this clinical situation may not preclude a successful SLN biopsy [20-22]. *Intra et al. *first reported on the feasibility of SLN biopsy in women with recurrent breast cancer following mastectomy and implant based reconstruction for previous DCIS [20]. In this small report of four patients, SLNs were identified in the axilla in all patients, two of whom had nodal metastasis. The remaining two patients did not have nodal dissection. No axillary recurrences were reported, but follow up was admittedly short. *Karam et al. *recently reported on 10-year experience from the Memorial Sloan Kettering Cancer Center with attempts at SLN biopsy in the setting of a previous mastectomy [21]. Of the 20 patients in whom it was attempted, 13 (65%) had successful SLN identification in the axilla, and nearly a 90% identification rate was observed in those treated previously with mastectomy and less than full axillary node dissection. Of the 13 patients with successful node identification, two were found to have metastasis to the SLN. Full axillary dissection was not performed on all patients, so false negative rates are not known; however, no patient had isolated axillary recurrences during follow up. Most recently, *Tasevski et al. *published a retrospective case series on SLN biopsy in18 patients with ipsilateral breast tumor recurrence after breast surgery; one of the two patients who underwent previous mastectomy and SLN biopsy had successful identification of a sentinel node, and it was negative for metastasis [22].

We cannot reliably speculate as to the origins of the lymphatic channels by which this recurrent cancer reached the axilla. It is possible that original lymphatic channels maintained in the superior flap of the mastectomy remained, that new collateralization of lymph channels had occurred, or perhaps that because of dermal involvement, that this recurrent cancer utilized sub-dermal lymphatics similar to that of a cutaneous malignancy. Whatever the pathway, it seems reasonable to assume that if lymph node metastases are to occur in cases of recurrent breast cancer, they will use the same pathway as that taken by sulfur colloid injection near the tumor. Peri-tumoral injection of radiocolloid therefore seems advisable when normal lymphatic anatomy has been profoundly disrupted. If preoperative lymphoscintigraphy maps to a nodal basin there is no reason to believe that SLN biopsy will not be successful.

National Comprehensive Cancer Network guidelines for breast cancer suggest that the management for cancer recurrence after mastectomy should entail resection with chest wall irradiation and individualized chemotherapy, but does not describe management of axillary nodes [23]. The findings resulting from SLN biopsy in our patient substantially affected her cancer treatment.

## Conclusion

Our report, in addition to those of others, suggests that SLN biopsy may be possible even after mastectomy. We advocate preoperative lymphoscintigraphy in such cases, and consideration of SLN biopsy if axillary nodal drainage is demonstrated. Based upon this report, we have initiated a prospective trial to identify sentinel node identification rates in this setting. If demonstrated to be feasible and reliable, sentinel lymph node biopsy after mastectomy may be an important addition to the armamentarium of surgical oncologists in treating patients with recurrent breast cancer.

## Consent

Written informed consent was obtained from the patient for publication of this case report and any accompanying images. A copy of the written consent is available for review by the Editor-in-Chief of this journal.

## Competing interests

The authors declare that they have no competing interests.

## Authors' contributions

DAV assisted with manuscript creation and editing, GH assisted with data collection, manuscript creation and editing, DS assisted with data collection and manuscript editing, PWS assisted in the study's concept and design, and manuscript editing, LRH assisted in the study's concept and design, data collection, and manuscript creation and editing. All authors read and approved the final manuscript.
